# A spatial and temporal transformer-based EEG emotion recognition in VR environment

**DOI:** 10.3389/fnhum.2025.1517273

**Published:** 2025-02-26

**Authors:** Ming Li, Peng Yu, Yang Shen

**Affiliations:** ^1^State Key Laboratory of Virtual Reality Technology and Systems, Beihang University, Beijing, China; ^2^Collaborative Innovation Center of Assessment for Basic Education Quality, Beijing Normal University, Beijing, China

**Keywords:** electroencephalograph, virtual reality, transformer, emotion recognition, brain-computer interface

## Abstract

With the rapid development of deep learning, Electroencephalograph(EEG) emotion recognition has played a significant role in affective brain-computer interfaces. Many advanced emotion recognition models have achieved excellent results. However, current research is mostly conducted in laboratory settings for emotion induction, which lacks sufficient ecological validity and differs significantly from real-world scenarios. Moreover, emotion recognition models are typically trained and tested on datasets collected in laboratory environments, with little validation of their effectiveness in real-world situations. VR, providing a highly immersive and realistic experience, is an ideal tool for emotional research. In this paper, we collect EEG data from participants while they watched VR videos. We propose a purely Transformer-based method, EmoSTT. We use two separate Transformer modules to comprehensively model the temporal and spatial information of EEG signals. We validate the effectiveness of EmoSTT on a passive paradigm collected in a laboratory environment and an active paradigm emotion dataset collected in a VR environment. Compared with state-of-the-art methods, our method achieves robust emotion classification performance and can be well transferred between different emotion elicitation paradigms.

## 1 Introduction

Affective Brain-Computer Interfaces (aBCIs) is a system that inducts, recognize, and regulate human emotions, which involves computer science, psychology, cognitive science, and more, aiming to enhance computers' ability to understand and respond to human emotional states during human-computer interaction (Tao and Tan, 2005). The Inputs of aBCIs typically include functional magnetic resonance imaging (fMRI), functional near-infrared spectroscopy (fNIRS), and electroencephalography (EEG). Among these, EEG non-invasively captures electrical activity on the scalp, offering high temporal resolution and relatively easy signal acquisition (Alarcao and Fonseca, 2017). Consequently, it has been widely applied in medical rehabilitation, education, and other fields (Zheng et al., 2023; Li et al., 2020). In the field of medical rehabilitation, aBCI can objectively and accurately assess emotional states, providing a valuable supplement to traditional diagnostic methods that are highly subjective, such as behavioral observations and questionnaires. In transportation, EEG signals can monitor drivers' negative emotional states like anxiety and anger in real time, providing timely warnings and adjustments to reduce traffic accidents (Zepf et al., 2020).

In these applications, emotion recognition is the most critical component (Hu et al., 2019). However, due to EEG signals' sensitivity to noise, numerous artifacts are introduced, posing significant challenges to emotion recognition. Traditional machine learning models extract features related to emotional states from EEG signals, such as power spectral density (PSD) (Solomon Jr, 1991) and differential entropy (DE) (Duan et al., 2013), and then feed them into classifiers like SVM (Wang et al., 2011), KNN (Mehmood and Lee, 2015), and MLP (Li et al., 2022a), achieving decoding of EEG. With the advancement of deep learning, researchers have developed various models to decode emotions from EEG, including supervised emotion recognition models based on convolutional neural networks (CNN) (Hwang et al., 2020), recurrent neural networks (RNN) (Alhagry et al., 2017), graph convolutional neural networks (GCN) (Song et al., 2018), and Transformers (Sun et al., 2022), which have made significant progress. CNNs perform well in classification tasks, particularly in fields like image (Bhatt et al., 2021), video (Xu et al., 2015), and speech processing (Hema and Marquez, 2023). RNNs excel at handling sequential data but face limitations in parallel training and global information capture (Ma et al., 2023). In contrast, Transformer models utilize self-attention mechanisms to effectively capture crucial long-term dependencies in time series (Chitty-Venkata et al., 2023).

Initially applied in natural language processing and computer vision with remarkable results, Transformers have recently begun to be employed in EEG encoding and decoding tasks (Abibullaev et al., 2023). By capturing long-term temporal relationships in EEG sequences, they extract robust feature representations. However, most studies focus solely on modeling either the temporal or spatial dimensions of EEG, allowing for the learning of relationships between different channels or time frames (Peng et al., 2023). When using a temporal-spatial Transformer, each channel of each frame is treated as a token. This approach leads to a significant increase in the number of tokens when processing long EEG sequences or multi-channel data, resulting in substantial computational demands.

Moreover, in the field of affective computing, emotional induction paradigms can be divided into laboratory settings (passive induction paradigms) and natural settings (active induction paradigms) (Meuleman and Rudrauf, 2021). However, current research is almost exclusively conducted in laboratory settings, where the passively induced emotional changes differ from the actively generated emotional changes in real-world scenarios (Miranda-Correa et al., 2018; Katsigiannis and Ramzan, 2017). Moreover, most emotion recognition studies have focused on training and testing models on a single dataset within a laboratory environment, with few studies validating the effectiveness of emotion recognition models in natural settings (Marín-Morales et al., 2020). Virtual reality (VR) can provide a highly immersive and realistic virtual environment, allowing for the assessment of emotional experiences in a more realistic scenario, making it an ideal paradigm for affective research (Marín-Morales et al., 2020).

In this paper, we conducted an emotional induction experiment in a VR environment and collected corresponding EEG data. We propose an emotion recognition method based on spatial and temporal Transformers (EmoSTT). EmoSTT employs two separate Transformers to model the temporal and spatial dimensions of EEG data, without being affected by the excessive number of tokens caused by long time sequences or multi-channel EEG data. The temporal and spatial Transformer blocks can learn the correlations between EEG time series and across channels, extracting hidden feature representations with spatial-temporal dependencies. Finally, the feature representations are fed into a simple fully connected layer to decode emotional states. Lastly, we validated the model's effectiveness on datasets of different emotional induction paradigms.

## 2 Related work

### 2.1 EEG-based emotion recognition

Traditional machine learning-based emotion recognition typically involves preprocessing, feature extraction, feature smoothing, training classifiers, and testing (Jenke et al., 2014). Signal features can be categorized into time-domain, frequency-domain, and spatial-domain features. Among these, frequency-domain features are most relevant to emotions, and DE features have been proven to offer the best emotion recognition performance (Jenke et al., 2014). It commonly use the Short Time Fourier Transform (STFT) to convert sequential EEG signals into the frequency domain, thereby extracting features from five frequency bands: δ band (1–3Hz), θ band (4–7Hz), α band (8–13Hz), β band (14–30Hz), and γ band (31–50Hz) (Mohammadi et al., 2017). Additionally, deep learning models are dedicated to designing neural networks that extract generalizable features. Li et al. (2016) further proposed a hybrid deep learning architecture (Convolutional and Recurrent Neural Network, C-RNN) for emotion recognition. The model extracts task-relevant features, explores channel correlations, and integrates contextual information across these frames, achieving excellent results on the DEAP dataset (Koelstra et al., 2011). Li et al. (2019) introduced a Spatial-Temporal Neural Network with Regional to Global (R2G-STNN) based on Bidirectional Long Short-Term Memory (BiLSTM), which conducts hierarchical feature learning from regional to global through spatial and temporal neural network models to extract discriminative spatiotemporal EEG features. Zhong et al. (2020) proposed a Regularized Graph Neural Network (RGNN) that considers the biological topology between different brain regions to capture local and global relationships between different EEG channels. Additionally, two regularizers were proposed, namely Node Domain Adversarial Training (NodeDAT) and Emotion-Aware Distribution Learning (EmotionDL), to better handle individual differences and noisy label issues. Song et al. (2022) proposed a novel compact convolutional Transformer network called EEG-Conformer for improving emotion recognition performance of EEG signals. EEG-Conformer combines convolutional modules to capture local temporal and spatial features, as well as self-attention modules to extract global correlations.

### 2.2 Active and passive emotion elicitation paradigms

However, the aforementioned methods are all trained and tested on data collected in traditional laboratory settings. These represent passive emotional changes, which differ from the active emotional changes that individuals generate in real-world scenarios, potentially leading to differences in EEG signals between these two paradigms (Somarathna et al., 2022). The ecological validity in emotional induction is crucial for affective research (Mohammadi and Vuilleumier, 2020). The passive emotional induction paradigm in traditional laboratory settings has weak induction effects (Soleymani et al., 2011). In contrast, virtual reality (VR) can simulate controlled environments with high immersion, presence, and interactivity, evoking emotions more naturally and authentically (Cao et al., 2021). Previous studies have validated the effectiveness of VR emotional induction paradigms (Li et al., 2022b). However, due to the lack of active paradigm emotional EEG datasets, research on emotion recognition models in active paradigms is very limited.

## 3 Methods

### 3.1 Pipeline

**Figure 1:**
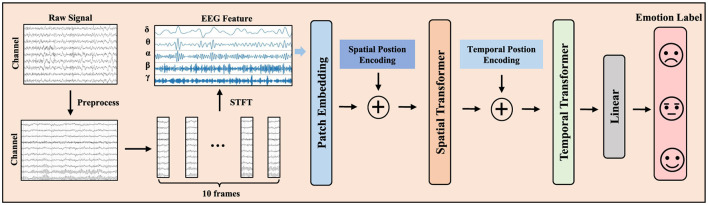
The overall model framework of EmoSTT.

The overall framework of the model is depicted in Figure 1. Initially, the raw EEG signals undergo preprocessing, followed by segmenting all signals into 1-second epochs. Consistent with (Duan et al., 2013), for each 1-second segment, we employ the Short-Time Fourier Transform (STFT) for frequency domain feature extraction. We extract the DE features from the EEG data of all participants **X** ∈ ℝ^*N*×*C*×*F*^ as the pre-training dataset to input our model, where N represents the number of samples in the preprocessed EEG dataset, C refers to the number of EEG channels, and F is the dimensionality of the DE features. Here, we set F = 5, corresponding to the five frequency bands: δ band (1–3Hz), θ band (4–7Hz), α band (8–13Hz), β band (14–30Hz), and γ band (31–50Hz). To better capture the temporal dimension of the EEG data, we use an overlapping time window of length *T* to transform the original signal **X** into the shape ${\text{X}}_{new}\in {\mathbb{R}}^{N_{new}\times T\times C\times F}$, which serves as the input for the final pre-training model, each sample is represented as ${\text{x}}_{s}\in {\mathbb{R}}^{T\times C\times F}$ Here, *N*_*new*_ represents the number of samples in the dataset. *T* denotes the number of time frames, which we set to 10, consistent with previous classical studies (Li et al., 2022c) to facilitate comparison. To mitigate the variability among features and enhance performance, we normalize training and testing data based on the mean and standard deviation of the training set. Subsequently, we utilize two separate Transformers to extract the spatial and temporal features of the EEG signals. Below is a detailed description of the method.

### 3.2 Spatial transformer

For the input to the spatial transformer ${\text{X}}_{new}\in {\mathbb{R}}^{N_{new}\times T\times C\times F}$, where each sample is represented as ${\text{X}}_{new}\in {\mathbb{R}}^{T\times C\times F}$. Then, we then project the frequency domain dimension K to a hidden dimension D using a linear projection matrix *P* ∈ ℝ^*k* × *D*^.

Since EEG signals record complex brain activities from multiple electrode channels, there is a strong correlation between different electrode channels. Therefore, using a spatial transformer can effectively encode the spatial information of EEG signals. For each given frame of data {${\text{x}}_{s}^{i}$ ∈ ℝ^*C* × *D*^ |i = 1, 2, ..., T}, where C is the number of channels, each channel is treated as a patch, and a learnable spatial position encoding ${\text{E}}_{SPos}\in {\mathbb{R}}^{C\times D}$ is added to ${\text{x}}_{s}^{i}$. This preserves the position information of each channel, which is crucial in transformers and is a standard and common practice. Here, we use learnable sine-cosine trigonometric functions as spatial position encoding. We utilize the attention mechanism to extract the functional connectivity relationships between different electrode channels by stacking multiple transformer blocks. The computation process of self-attention is as follows:


(1) $$\begin{array}{l} \mathrm{Attention}\left(Q,K,V\right)=\mathrm{Softmax}\left(\frac{QK^{T}}{\sqrt{d_{k}}}\right)V \end{array}$$

**Figure 2:**
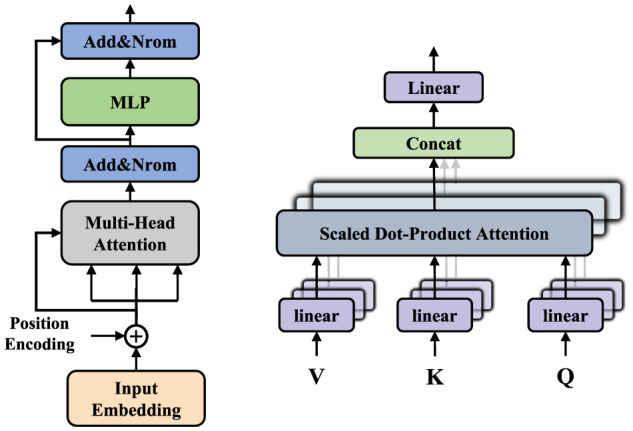
The left is the overall architecture of the transformer, and the right is the multi-head attention module.

In this context, *Q*, *K*, and *V* represent the query, key, and value, respectively. For each input vector, three linear transformations are applied to map it into the query vector, key vector, and value vector. For each query vector, the similarity to all key vectors is calculated using a dot product, resulting in attention scores. To prevent the dot product from becoming too large, it is divided by the square root of *d*_*k*_; subsequently, the Softmax function is applied to normalize the results of the aforementioned dot product. Finally, after obtaining the Softmax matrix, it is multiplied by *V* to produce the final output. Here, we utilize the multi-head attention mechanism (MHA), which captures dependencies in different subspaces of the input sequence by computing multiple sets of queries, keys, and values in parallel. The outputs of these heads are then concatenated and passed through a linear transformation to yield the final output. As illustrated in Figure 2, in addition to the multi-head attention mechanism, each Transformer encoder includes multi-layer perceptrons (MLPs), with each component employing residual connections and layer normalization to enhance the model's training efficiency and performance.

### 3.3 Temporal transformer

Subsequently, the output from the spatial Transformer module is fed into the temporal encoder module. We add the same sine-cosine positional encoding to keep track of the position of each time frame. Like the spatial Transformer, we stack the same number of Transformer blocks. Unlike RNN, transformers are better at capturing long-term dependencies in EEG signals. The encoder also includes multi-head attention (MHA) and multi-layer perceptrons (MLPs). Finally, for the output of the temporal Transformer, we pass it through a simple MLP block with layer normalization and a linear layer to obtain the final classification output **y** ∈ ℝ^*C*×*K*^. In this paper, we use cross-entropy loss to minimize the error between the predicted emotion categories and the true emotion labels:


(2) $$\begin{array}{l} L=-\frac{1}{N}\overset{N}{\underset{n=1}{\sum }}\overset{K}{\underset{k=1}{\sum }}y_{n}^{k}\log\left({\hat{y}}_{n}^{k}\right) \end{array}$$

where *N* represents the number of batch sizes and *K* represents the number of categories. $y_{n}^{k}$ is the true emotion label and ${\hat{y}}_{n}^{k}$ is the predicted emotion category.

## 4 Experiments

### 4.1 Dataset

This paper validates the proposed method on a widely used public dataset, SEED (Zheng and Lu, 2015), as well as on a self-collected VR emotion dataset (VR-Emotion). Both datasets consist of EEG signals collected from participants while they watch video stimuli in a quiet laboratory or a VR environment. After viewing the video stimuli, participants provide self-assessments of their emotional responses, which serve as the labels for the EEG data. Below is a brief introduction to the datasets:

#### 4.1.1 SEED dataset

The SEED dataset primarily comprises EEG signals corresponding to three types of emotions: positive, neutral, and negative. The data were collected using a 62-channel device from the ESI NeuroScan system, with a sampling rate of 1,000 Hz. To account for the impact of cultural differences on emotion recognition research, all movie clips selected for this dataset are in Mandarin. The three emotion experiments involved 15 participants (7 males and 8 females), all Chinese students from Shanghai Jiao Tong University. To verify the stability of emotions over different periods, each participant experimented three times, with a one-week interval between each session, resulting in a total of 45 experiments. Participants were required to watch 15 emotional stimulus videos in each experiment, corresponding to the three emotion categories. Each video ranged from 185 to 238 seconds in length. Each second of EEG data corresponding to the video is considered as one sample, so there are 3394 samples in each session.

#### 4.1.2 VR-emotion dataset

In previous experiments, we have verified the effectiveness of the VR emotion induction paradigm (Li et al., 2022b). We recorded EEG signals from participants using the ANT Neuro EEG acquisition system while they watched VR video stimuli. The following principles guided the selection of VR video stimuli: (1) Each video should not be too long to avoid causing mental fatigue in participants; (2) The content must be clear and understandable without the need for translation; (3) The movie clips must evoke a single target emotional state. Therefore, we selected 4 videos from the Stanford public VR video dataset [2 for low valence/low arousal (LVLA) and 2 for high valence/low arousal (HVLA)] (Li et al., 2017). Due to the lack of high arousal/low valence (HALV) videos, we chose 15 of YouTube's most viewed horror videos. We then invited 16 students majoring in psychology from Beijing Normal University to rate the videos' emotional arousal and valence dimensions. For each video *x*, we calculated the normalized arousal and valence scores by dividing the average score by the standard deviation (μ_*x*_/σ_*x*_). Ultimately, we selected two horror videos with extreme angles in the VA plane quadrant: “Real Run” and “The Conjuring 2.” For the high arousal/high valence (HAHV) videos, during the pre-experiment, all participants reported severe motion sickness from these two videos, which could affect EEG analysis (Jeong et al., 2019). To eliminate the impact of motion sickness in the experiment, we chose the VR game “The Blu” from the Steam platform. This game offers a more passive and intuitive experience, akin to watching a video, and has been proven to evoke high arousal/positive valence emotions while providing an immersive VR experience (Meuleman and Rudrauf, 2021). The game includes three segments: “The Reef Migration,” “The Whale Encounter,” and “The Hammerhead Cove.” We invited the same psychology students to evaluate these three segments and ultimately selected “The Whale Encounter” and “The Reef Migration” as the final emotional stimulus materials.

The dataset comprises EEG data from 28 participants (16 males and 12 females) collected while viewing 8 VR videos, using a 32-channel EEG system with a sampling rate of 512 Hz. Each video is approximately 3 min and 30 seconds long. EEG data corresponding to every second of each video is considered a sample, amounting to 1,483 samples per participant.

### 4.2 EEG preprocessing and feature extraction

The authors provided the original preprocessed DE features for the SEED dataset, which we directly use as input in this chapter. As for the VR-Emotion dataset, to obtain clean and high-quality EEG signals, we require participants to avoid excessive head movements while recording EEG signals in VR to ensure signal stability and reliability. To prevent the HMD from exerting pressure on the front-central electrodes, a lateral elastic band is used to fix the HMD while the upper elastic band is loose. Additionally, to avoid the potential impact of pressure from repeatedly wearing the VR headset on the quality of the EEG signals, the subjective questionnaire is presented directly on the VR screen, enabling participants to complete it without removing the headset. For the raw EEG data, we employ EEGLAB for EEG signal processing (Delorme and Makeig, 2004). EEGLAB is an open-source Matlab toolbox that provides algorithms for EEG preprocessing and feature extraction. The specific steps are as follows: (1) First, the original EEG data has a sampling frequency of 512 Hz, which is sufficient to filter out interference from the monitor (50–60 Hz) and the VR headset (90 Hz). The signals are then downsampled to 128 Hz and re-referenced using bilateral mastoid electrodes (M1 and M2). (2) Since EEG signals are low-frequency and electromyographic artifacts are high-frequency, low-pass filtering removes the EMG artifacts significantly. Furthermore, a bandpass filter (4–47 Hz) is applied to the signal using the FIR filter in EEGLAB to filter out eye movement artifacts better. (3) Visual inspection is performed to remove abnormal signals with amplitudes exceeding ±100 μV, as signals beyond this threshold are considered non-EEG signals. (4) Independent Component Analysis (ICA) (Chaumon et al., 2015) is then used to decompose the original signal into 32 independent components (ICs). ICA is a method based on Blind Source Separation (BSS). Using the SASICA plugin in EEGLAB and visual inspection, we identify which components are related to emotion and which are artifacts or other neural activity components (such as eye blinks, muscle activity, or head movements). (5) Finally, for each participant, an average of 9.37 components are removed, yielding clean EEG signals.

Then, we extract DE features based on the preprocessed data in the same way as the previous studies (Duan et al., 2013). DE has been proven to be one of the most effective features for emotion recognition (Qiu et al., 2024; He et al., 2022). DE features are an extended form of Shannon's information entropy $-\underset{x}{\sum }p\left(x\right)\log\left(p\left(x\right)\right)dx$ for continuous variables, and they are calculated as follows:


(3) $$\begin{array}{l} DE=-\int_{a}^{b}p\left(x\right)\log\left(p\left(x\right)\right)dx \end{array}$$

In this context, *p*(*x*) represents the probability density function of continuous information, and |*a, b*| indicates the interval over which the information is taken. For a segment of EEG signals that approximately follow a Gaussian distribution $N\left(\mu ,\sigma_{i}^{2}\right)$ for a specific length, its differential entropy equals the logarithm of its energy spectrum in a particular frequency band.


(4) $$\begin{array}{r} DE=-\int_{-\infty }^{\infty }\frac{1}{\sqrt{2\pi \sigma_{i}^{2}}}e^{-\frac{{\left(x-\mu \right)}^{2}}{2\sigma_{i}^{2}}}\log\left(\frac{1}{\sqrt{2\pi \sigma_{i}^{2}}}e^{-\frac{{\left(x-\mu \right)}^{2}}{2\sigma_{i}^{2}}}\right)\\ dx=\frac{1}{2}\log\left(2\pi \;e\sigma_{i}^{2}\right) \end{array}$$

### 4.3 Evaluation

To thoroughly evaluate the effectiveness of the emotion recognition pre-training framework, this chapter conducts subject-specific emotion recognition experiments on the SEED and VR-Emotion datasets, that is, training an emotion classification model separately for each individual's EEG data from each experimental session. Consistent with previous research methods (Zheng et al., 2018; Liu et al., 2021), the training, fine-tuning, and testing data come from a single session on the same subject. In the SEED dataset, the first 9 trials of each session are used as training data (with 3 trials for each emotion category to maintain data balance), and the last 6 trials are used as testing data. Consequently, there are a total of 45 emotion recognition models, and the final classification accuracy is defined as the average accuracy obtained from these 45 models. For the VR-Emotion dataset, the first 6 trials of each subject are used as the training set (3 for high arousal/low arousal and 3 for low valence/high valence, to maintain data balance), and the last 2 trials are used as the test set. Thus, there are a total of 28 emotion recognition models, and the final classification accuracy is defined as the average accuracy obtained from these 28 models.

### 4.4 Implementation

This paper employs the PyTorch framework to train models on an NVIDIA 4090 GPU. The model utilizes four Transformer modules, with the number of attention heads set to 8 and the hidden dimension *D* set to 16. We adopt an exponential learning rate decay scheme with an initial learning rate of 2 × 10^−4^ and a decay factor of 0.98 per epoch. Weight decay and batch size are set to 0.1 and 128, respectively. The model is optimized using the AdamW optimizer. For both temporal and spatial encoder, we use *L* = 6 transformer blocks. The embedding dimension *D* is set to 32, and the multi-head number *H* is set to 6.

### 4.5 Results

#### 4.5.1 SEED dataset

We validate the model's classification results on the SEED dataset using topic-related experiments. As shown in the Table 1, we compared our model with several other supervised models. The results indicate that our model achieved an accuracy of 92.67% and an F1 score of 86.78% for positive, negative, and neutral emotions, outperforming other supervised methods. This demonstrates that the model can extract robust and highly discriminative features.

**Table 1:** The ACC/F1 of subject-dependent experiments on the SEED dataset.

**Method**	**SVM**	**RF**	**DBN**	**GRSLR**	**GCNN**
**ACC/F1**	83.99/78.93	78.46/76.58	86.08/79.89	87.39/80.74	87.40/81.33
**Method**	DGCNN	DANN	BiDANN	EmoSTT	
**ACC/F1**	90.40/84.85	91.36/85.26	92.38/86.28	**92.67/86.78**	

Bold indicates that our method achieved the best results.

**Figure 3:**
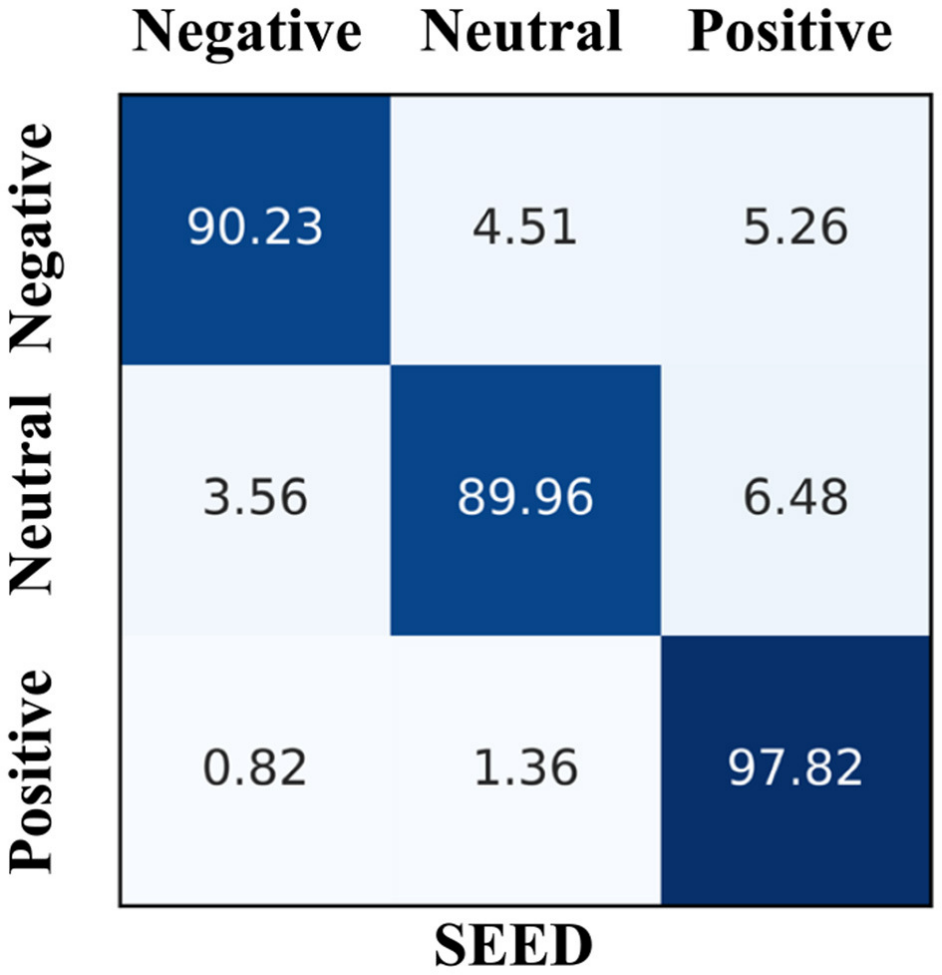
The confusion matrix of SEED dataset classification results.

Figure 3 presents the confusion matrix of EmoSTT's results on the SEED dataset, where each row represents the true class and each column represents the predicted class. The results indicate that positive emotions are the most easily identified, achieving an accuracy rate of 97.82%. The accuracy rates for neutral and negative emotions are 89.96% and 90.23%, respectively. Additionally, we observe that neutral and positive emotions are more likely to be confused, a mix-up that may stem from the difficulty participants face in distinguishing between these two emotions during the emotion induction process.

In addition, we also list the average accuracy of three experiments for each subject and compare them with DGCNN (Song et al., 2018), as shown in the Table 2. It can be seen that the model achieves a relatively high average accuracy on the 15 subjects of the SEED dataset, and the average accuracy of our proposed EmoSTT is higher.

**Table 2:** Comparison of accuracy with standard deviation between DGCNN and EmoSTT.

**Subject**	**DGCNN**	**EmoSTT**
1	89.39 ± 0.93	96.72 ± 6.8.67
2	80.00 ± 1.95	88.92 ± 8.21
3	83.85 ± 1.34	96.47 ± 6.54
4	94.01 ± 1.87	92.94 ± 7.24
5	85.12 ± 1.09	88.07 ± 5.64
6	91.45 ± 0.78	90.95 ± 7.51
7	91.45 ± 1.41	96.10 ± 5.89
8	87.77 ± 1.74	91.72 ± 7.67
9	94.37 ± 1.66	92.35 ± 6.53
10	82.99 ± 1.50	79.06 ± 7.98
11	92.10 ± 0.67	95.58 ± 6.83
12	90.04 ± 1.99	92.68 ± 7.09
13	90.66 ± 1.04	93.63 ± 6.46
14	92.60 ± 1.25	95.29 ± 8.29
15	97.79 ± 0.46	99.49 ± 7.85
Aver	90.40/08.49	92.67/7.05

#### 4.5.2 VR-emotion dataset

The results of the VR-Emotion dataset are shown in Table 3. EmoSTT achieved accuracy of 75.67% and 76.47% in the arousal and valence dimensions, respectively. The corresponding F1 scores are 70.83% and 71.29%, respectively. It can be seen that the model can also maintain robust performance under the active emotion induction paradigm. For emotional arousal, it outperformed SVM, RF, and DGCNN by 11.09%, 9.19%, and 3.21%, respectively. For emotional valence, it surpassed SVM, RF, and DGCNN by 8.11%, 7.05%, and 4.88%, respectively.

**Table 3:** Comparison of models on VR-emotion dataset for arousal and valence.

**Model**	**VR-emotion (arousal)**	**VR-emotion (valence)**
SVM	64.58/60.34	68.36/64.21
RF	66.48/61.38	69.42/65.46
DGCNN	72.48/67.18	71.59/67.73
EmoSTT	75.67/70.83	76.47/71.29

The result is expressed as ACC/F1.

**Figure 4:**
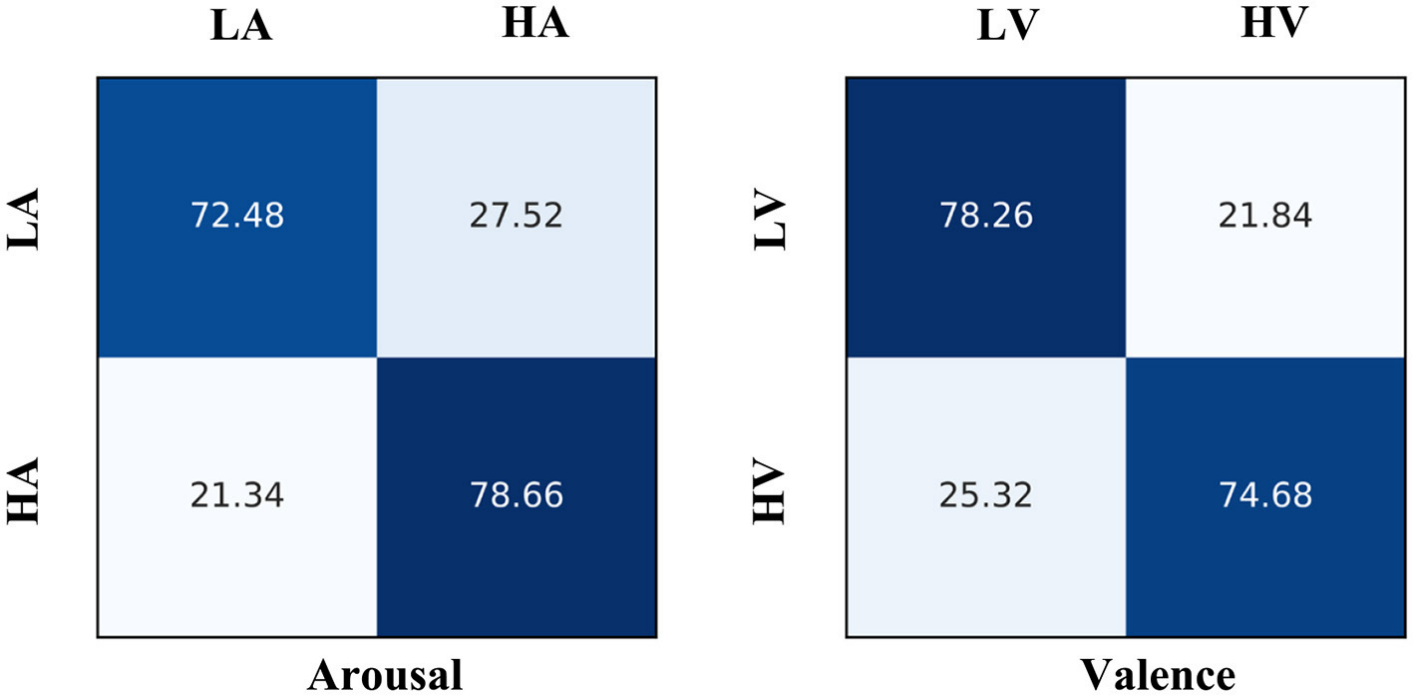
The confusion matrix of arousal and valence classification results of VR-emotion dataset.

For the VR-Emotion dataset, Figure 4 shows the confusion matrix of the EmoSTT classification results. It can be seen that high-arousal emotions are relatively easy to identify, reaching an accuracy of 78.66%, which is 6.18% higher than the recognition of low-arousal emotions. We speculate that this is because VR videos are more advantageous in inducing high-arousal emotions. In terms of valence, the recognition accuracy of low valence is 78.26%, which is 3.58% higher than the 74.68% of high valence.

## 5 Conclusion and future work

In this paper, we propose EmoSTT, an emotion recognition model based on a pure Transformer model that can extract temporal and spatial dependency features of EEG signals. Taking advantage of the high ecological validity of the VR emotion induction paradigm, we collect an emotional EEG dataset of subjects watching VR videos. The performance of EmoSTT is verified on datasets of two different emotion induction paradigms. The model achieves an accuracy of 92.67% on the SEED passive induction dataset, and 75.67% and 76.47% arousal and valence classification accuracies on the VR-Emotion dataset, respectively. The results show that the model can well transfer the emotion recognition model in the laboratory environment to natural environments such as VR. In the future, we will validate our approach in a broader range of scenarios. This includes using more natural VR interactive environments, ensuring signal stability, and verifying the effectiveness of emotion induction as well as the robustness of the model.

## Data Availability

The data analyzed in this study is subject to the following licenses/restrictions: this dataset is an emotional EEG dataset. Requests to access these datasets should be directed to minglee@buaa.edu.cn.
